# Identification and characterization of *Staphylococcus* spp. Isolated in co-infection with respiratory viruses from children in ICUs

**DOI:** 10.1016/j.bjid.2025.104591

**Published:** 2025-10-24

**Authors:** Kelliane Martins de Araujo, Marcos de Oliveira Cunha, Vivian Maria Cordeiro, Angélica de Lima das Chagas, José Daniel Gonçalves Vieira, Celia Regina Malveste Ito, Thais Reis Oliveira, Lucas Candido Gonçalves Barbosa, Isabela Wastowski Jubé, Lilian Carla Carneiro

**Affiliations:** a Universidade Federal de Goiás, Institute of Tropical Pathology and Public Health, Microorganism Biotechnology Laboratory - LBMIc, Goiânia, GO, Brazil; b Universidade Federal de Goiás, Institute of Tropical Pathology and Public Health, Laboratory of Environmental Microbiology and Biotechnology - LAMAB, Goiânia, GO, Brazil; c Universidade Estadual de Goiás, Molecular Immunology laboratory, Goiânia, GO, Brazil

**Keywords:** Bacterial co-infection, COVID-19, Bacterial resistance, Infection, Epidemiology

## Abstract

Healthcare-associated infections are among the most significant complications in hospitalized patients, posing a major challenge due to the antimicrobial resistance of pathogenic agents such as *Staphylococcus* spp. The study aims to identify and evaluate the phenotypic and molecular resistance profile of *Staphylococcus* spp. in co-infection with respiratory viruses, including COVID-19, as a respiratory virus, in samples from children admitted to ICUs. Nasopharyngeal samples from the biorepository were stored at -80 °C in medium containing gentamicin and amphotericin B. Bacterial strains were isolated, and antibiograms were performed using the Kirby-Bauer method with antimicrobials specific to *Staphylococcus* spp. and the method of evaluating molecular resistance, carrying out the amplification of resistance genes, using specific oligonucleotides. A multidrug-resistant profile was observed in *Staphylococcus* spp., highlighting the need for monitoring to ensure appropriate treatment. Antimicrobial resistance emphasized the importance of strict control over antibiotic use in hospital environments. This study contributes to the understanding of antimicrobial resistance in bacterial co-infections, providing insights for more effective treatments and HAI control strategies.

## Introduction

Healthcare-Associated Infections (HAIs) are a global public health problem, challenging professionals, managers and researchers due to their high morbidity and mortality rates and financial impact. HAIs occur in healthcare settings, such as hospitals, clinics and intensive care units, due to invasive practices, immunosuppressed patients and failures in infection control protocols.^1^

Antimicrobial resistance is one of the biggest problems in the context of HAIs. Microorganisms such as Methicillin-Resistant *Staphylococcus aureus* (MRSA), *Escherichia coli* and *Klebsiella pneumoniae* are commonly associated with these environments, developing resistance to broad-spectrum antibiotics due to prolonged and, in some cases, inappropriate use of these drugs.^2^

During the COVID-19 pandemic, the development of Antimicrobial Resistance (AMR) was mainly driven by the widespread and inappropriate use of antibiotics. Coronavirus is a viral disease; however, this has not been an obstacle to the prescription of antibiotics for patients with COVID-19, despite their ineffective action against viruses.^3^, ^4^, ^5^, ^6^

It is estimated that, if nothing is done, antimicrobial resistance could cause up to 10 million deaths annually by 2050, surpassing the number of deaths caused by cancer. In addition, bacterial resistance could impose a significant economic cost, with loss of productivity and increased health care costs, mainly due to prolonged and more expensive treatments required to combat resistant infections.^7^

Studies have shown that early detection of the *bla*Z, *mec*A and *fem*A genes allows for better control of hospital infections and a significant reduction in mortality associated with infections caused by resistant *Staphylococcus* spp.^8^

Identify *Staphylococcus* spp. in co-infection with respiratory viruses, including COVID-19, by evaluating the phenotypic and molecular resistance profile in samples from pediatric ICU patients.

## Materials and methods

### Origin of samples

All biological material for this proposal came from the biorepository of the project entitled: “Differential diagnosis and pediatric clinical evolution of COVID-19 in the context of seasonality of respiratory viruses in a capital city in the Midwest of Brazil”, approved by CEP/HC under protocol number: CAAE: 33540320.7.0000.5078. The samples for the study of the viral panel are stored in the MIV viral transport medium, refrigerated at −80 °C. A total of 150 samples previously collected in neonatal ICUs of five hospitals in the city of Goiânia were included in this study.

### Bacterial activation and identification

The bacterial strains associated with the viral panel of this study were initially seeded in BHI broth with a sterilized loop for reactivation and incubated for 24 h in a bacteriological incubator at 37 °C. The samples that presented turbidity in the BHI broth were seeded in nutrient agar for characterization and isolation of colonies and subsequent Gram staining. Those that presented gram-positive cocci morphology were followed for identification and extraction of chromosomal and plasmid DNA.

### Identification of bacterial species via MALDI-TOF spectrometry

With the bacterial colonies properly isolated, the samples were identified using the Matrix-Assisted Laser Desorption Ionization Mass Spectrometry with Time-of-Flight Analysis (MALDI-TOF) system.

### Antimicrobial susceptibility testing

The antibiogram was performed according to the Clinical and Laboratory Standards Institute (CLSI) guide for interpreting the Minimum Inhibitory Concentration (MIC) and halo diameters. Version 2024 for *Staphylococcus* spp. strains. The Kirby & Bauer (1966) methodology was applied.^9^ The antibiotic disks used were from BCLabor®: Clindamycin (2 µg); Rifampicin (5 µg); Levofloxacin (5 µg); Amikacin (30 µg) and Cefoxitin (30 µg). ATCC 25,923 was used as a negative control for this test.

### Extraction of chromosomal DNA

For the extraction of chromosomal DNA from *Staphylococcus* spp., the PureLink™ Mini Kit for Genomic DNA (THERMO FISHER SCIENTIFIC, 2012) was used.

### Extraction of plasmid DNA

For the extraction of plasmid DNA, the protocol used was developed in-house, with the two strains of *Staphylococcus* spp. inoculated in BHI at 37 °C for 24 h under agitation. After cultivation, the material was centrifuged three times to concentrate the cells, discarding the supernatant. The cell pellet was resuspended in 200 µL of a buffer solution I containing RNase, followed by the addition of 200 µL of solution II, mixing by inversion for 5-minutes to promote cell lysis. Then, 200 µL of solution III were added for neutralization, homogenizing the sample for another five minutes.

After centrifugation, the supernatant containing the plasmid DNA was transferred to another tube and treated with isopropanol to precipitate the DNA, leaving it to stand for 10 min at room temperature. The sample was centrifuged again and the supernatant discarded. The precipitate was allowed to dry and then resuspended in 30 µL of sterile distilled water, obtaining the purified plasmid DNA for quantification. The samples were then evaluated for extract quality by quantification in a GE Healthcare NanoVue™ device. Each sample was quantified individually, using sterile milli-Q water as a blank to ensure accurate calibration between samples.

### Characterization of the genotypic resistance profile

Table 1 lists the specific oligonucleotides used to amplify resistance genes and RNA16S for *Staphylococcus* spp.

**Table 1:** Oligonucleotides used to amplify resistance genes.

Genes	Gene sequence 5′ to 3′	Quantity of bases	References
*mec*A	F: 5′-AAAATCGATGGTAAAGGTTGGC-3′	533 pb	^10^
	R: 3′-AGTTCTGCAGTACCGGATTTGC-5′		
*blaZ*	F: 5′-AAGAGATTTGCCTATGCTTC-3′	517 pb	^11^
	R: 3′-GCTTGACCACTTTTATCAGC-5′		
*femA*	F: 5′-AAAAAGCACATAACAAGCG-3′	132 pb	^12^
	R: 3′-GATAAAGAAGAAACCAGCAG-5′		
RNA 16S	F: 5′-TGGCATAAGAGTGAAAGGCGC-3′	178 pb	^13^
	R: 3′-GGGGACGTTCAGTTACTAACGT-5′		

bp, base pairs; F, Forward; R, Reverse.

The reactions were prepared using the Sybr Green LGC Biotechnology Real-Time Polymerase Chain Reaction (PCR) kit (Sybr Green qPCR master mix LOW ROX – 100 reactions ×25 µL). The reaction and cycling conditions followed the manufacturer's instructions. The negative control was added with Milli Q water instead of DNA.

### Data analysis

To verify the association and correlation between phenotypic resistance, molecular findings, clinical signs and patient outcome using Descriptive Analysis, Analysis of Variance and correlation tests in the STATISTICA software version 7.0 (StatSoft©) (StatSoft I, 2012).

## Results

A total of 288 samples were collected from five hospitals with neonatal ICUs in the city of Goiânia, state of Goiás. Of the 288 samples collected, 99 tested positive for 16S RNA (presenting bacterial co-infection) and were included in the study. From these positive samples, 176 bacterial strains were isolated, identified and confirmed as genus and species; noting that each sample contained two or even three distinct species in co-infection using the MALDI-TOF technique. Of the 176 bacterial species identified, 34 (19.32 %) were from the genus *Staphylococcus* spp. *Staphylococcus lugdunensis* was responsible for 21 (11.9 %) of the cases, *Staphylococcus epidermidis* was identified in 6 (3.4 %) of the cases, *Staphylococcus aureus* in 5 (2.8 %), and *Staphylococcus haemolyticu*s in 2 (1.1 %), the remaining 142 strains (80.68 %) were classified as belonging to other genera (Table 2).

**Table 2:** Description in absolute and relative values of the bacteria found in samples by MALDI-TOF.

Bacteria	Absolute value	Relative Value ( %)
*Staphylococcus lugdunensis*	21	11.93
*Staphylococcus epidermidis*	6	3.41
*Staphylococcus aureus*	5	2.84
*Staphylococcus haemolyticus*	2	1.14
Other	142	80,68
**Total**	**176**	**100**

All strains of *Staphylococcus* spp. showed growth in nutrient agar culture medium after the bacterial activation process. However, during the phenotyping period, six samples were lost, and another eight samples did not have complete biochemical tests and unfortunately, we no longer have the original samples resulting in 20 isolates for final analysis. The species are highlighted in Table 3: *Stapylococcus* spp. strains were collected from nasopharyngeal swab samples of 20 pediatric patients, including 12 females and 8 males, with ages ranging from 0 to 84 months (mean = 34.6 months; median = 30 months). Viral detection rates were positive for different respiratory pathogens: SARS-like viruses in 10 % of cases, COVID-19 in 30 %, rhinovirus in 25 %, and RSV in 25 %. Less frequent detections included Bocavirus (5 %) and Cov NL63 (5 %).

**Table 3:** Total samples of the genus *Staphylococcus* spp.

Gender	Quantity	Percentage
*Staphylococcus aureus*	3	15 %
*Staphylococcus epidermidis*	5	25 %
*Staphylococcus haemolyticus*	1	5 %
*Staphylococcus lugdunensis*	11	55 %
Total	20	100 %

Regarding clinical manifestations, flu syndrome was the most common (45 %), followed by pneumonia (30 %). Less frequent conditions included cough, ENCP, myocarditis, congenital malformation, laryngitis, asthmatic attack, genetic syndrome, MISC, and bronchiectasis, each occurring in up to 5 % of cases. Length of hospital stay ranged from 0 to 60 days, with a median of 5.5 days and a mean of 10.8 days. Mortality was recorded in 5 % of the patients (*n* = 1).

As for therapeutic interventions, all patients received Oseltamivir and Salbutamol, 90 % were treated with corticosteroids, and 60 % received antibiotics. In addition, all patients had a documented history of family members with COVID-19, suggesting a potential exposure pathway.

### Morphological and physiological characteristics

Phenotypic tests provided an initial and complementary basis for the identification of *Staphylococcus* spp. Initially, advanced methods, such as MALDI-TOF, were not easily accessible in the first stage of research development. Morphological observation and specific biochemical tests such as catalase, coagulase, DNase, among others, constituted valuable tools for the recognition of the genus and species of Staphylococcus spp.

The morphological results found allowed us to observe some of the most common phenotypic characteristics among *Staphylococcus* spp. species. The circular shape and convex elevation of the colonies, for example, are aspects that predominated in the phenotypic tests for most of the *Staphylococcus* spp. samples and coincide with the classic descriptions for the genus. However, variations indicate the presence of less common species or lineage variants. Certain *Staphylococcus* species, such as *Staphylococcus aureus*, tend to exhibit specific characteristics that distinguish them from coagulase-negative species, such as *Staphylococcus epidermidis*.

The results found for the evaluation of the Gram test of the genus, exhibit a morphologically predominant profile in the form of gram-positive cocci, organized in clusters.

The species of greatest clinical relevance stand out: *S. aureus, S. epidermidis, S. haemolyticus* and *S. lugdunensis*, where most of the species found, with the exception of *S. lugdunensis*, consistently present in the form of gram-positive cocci, while *S. lugdunensis* can occasionally assume the coccobacillary form, although it remains gram-positive.

In complementing the identification, other phenotypic tests, such as Catalase, Hemolysis and Coagulase, were essential to differentiate the *Staphylococcus* spp. species and confirm their pathogenic and ecological characteristics, as shown in Table 4.

**Table 4:** Biochemical tests of the genus *Staphylococcus* spp.

Species	Catalase	Hemolysis	Coagulase
*Staphylococcus aureus*	+	Gama	-
*Staphylococcus epidermidis*	+	Beta	+
*Staphylococcus haemolyticus*	+	Alfa	-
*Staphylococcus lugdunensis*	+	Beta	+

(+) Positive test; (-) Negative test.

### Biochemical tests

Biochemical tests in microbiology are essential methods for identifying and characterizing microorganisms, especially bacteria, based on their metabolic and enzymatic reactions. They allow analysis of the microorganisms' ability to use certain substrates and produce certain metabolic products.

The results of biochemical tests, including Glucose, Maltose, Lactose, Sucrose, Urea and Novobiocin, confirmed the presence of *Staphylococcus* spp. in the samples, differentiating them from other Gram-positive cocci genera. Among these tests, the Novobiocin test stands out, a laboratory procedure used to identify different species of the genus *Staphylococcus* spp. This method can be used to determine whether a bacterium is sensitive or resistant to novobiocin, a type of antibiotic. Among the twenty samples analyzed, the Novobiocin biochemical test revealed that 35 % of them were resistant, while the other 65 % were sensitive. The vast majority of samples tested positive for glucose, sucrose, maltose and lactose. Other species identified, such as *Staphylococcus epidermidis* and *Staphylococcus aureus*, tested negative in the biochemical test for urea. On the other hand, species such as *Staphylococcus haemolyticus* and *Staphylococcus lugdunensis* tested positive for urea.

### Antibiogram

The antibiogram is a laboratory test that assesses the susceptibility of bacteria to different antimicrobials. The chosen antimicrobials were Clindamycin 2 µg (Class: Lincosamides), Rifampicin 5 µg (Class: Rifamycins), Levofloxacin 5 µg (Class: Fluoroquinolones), Amikacin 30 µg (Class: Aminoglycosides) and Cefoxitin 30 µg (Class: Cephalosporins, second generation) due to their characteristics and efficacy against different strains of *Staphylococcus* spp., including *Staphylococcus aureus* and other coagulase-negative Staphylococci. The results on sensitivity and resistance of the samples for each antibiotic class are shown in Table 5.

**Table 5:** Results on sensitivity and resistance of the samples for each antibiotic class.

ID	MALDI-TOF	CLI	RIF	LEV	AMI	CFO
		2 µg	5 µg	5 µg	0 µg	30 µg
1	*S. epidermidis*	S	S	**R**	S	**R**
2	*S. aureus*	S	S	S	S	S
3	*S. epidermidis*	S	S	I	S	**R**
4	*S. aureus*	S	S	S	S	S
5	*S. epidermidis*	S	S	S	S	S
6	*S. epidermidis*	**R**	S	S	**R**	**R**
7	*S. epidermidis*	**R**	S	S	**R**	**R**
8	*S. haemolyticus*	**R**	S	I	**R**	**R**
9	*S. aureus*	S	S	S	S	S
10	*S. lugdunensis*	S	S	I	**R**	S
11	*S. lugdunensis*	S	S	I	**R**	S
12	*S. lugdunensis*	**R**	**R**	**R**	**R**	**R**
13	*S. lugdunensis*	**R**	**R**	**R**	**R**	**R**
14	*S. lugdunensis*	**R**	R	I	**R**	**R**
15	*S. lugdunensis*	**R**	**R**	I	**R**	**R**
16	*S. lugdunensis*	S	S	**R**	**R**	S
17	*S. lugdunensis*	S	S	I	**R**	S
18	*S. lugdunensis*	S	S	I	**R**	S
19	*S. lugdunensis*	S	S	I	**R**	S
20	*S. lugdunensis*	S	S	I	**R**	S

CLI, Clindamycin; RIF, Rifampicin; LEV, Levofloxacin; AMI, Amikacin; CFO, Cefoxitin; S, sensitivity; **R**, Resistant; I, Indeterminate.

During the research development process, at the antibiogram stage, 14 samples were lost, reducing the total to twenty samples of the genus *Staphylococcus* spp. to be studied. To better discriminate the findings, the samples were identified by an identification number (ID). All 20 isolates of *Staphylococcus* spp. were subjected to susceptibility testing to four antimicrobials using the disk diffusion method.

The results of the antibiogram confirmed that several species of *Staphylococcus* spp., such as *S. epidermidis, S. aureus*, and *S. haemolyticus*, presented considerable variability in resistance profiles. *S. epidermidis* presented broad resistance to antibiotics such as Clindamycin and Cefoxitin in approximately 40 % and 80 % of the samples, respectively, while *S. aureus* demonstrated high sensitivity to the antibiotics tested. The bacteria were identified and were done with antibiogram testing show that the 30 % of the bacteria were resistance to clindamycin tasted, 20 % of samples were resistant to rifampicin and levofloxacin and one sample show intermediate resistance to rifampicin, on the other hand 70 % of the samples were resistance to amikacin and 50 % of the samples studied were resistant to cefoxitin.

Of the 20 *Staphylococcus* spp. isolates, resistance was observed to some of the antimicrobial classes described below. Cefoxitin was the antimicrobial with the highest percentage of resistance, with 55 % of the samples being resistant, followed by Amikacin, with 50 % of the isolates being resistant. The percentage of resistance to Amikacin (50 %) is particularly worrying due to its significant impact on the effectiveness of antimicrobial treatments.

The results demonstrated significant variability in antimicrobial susceptibility. While *S. aureus* showed high sensitivity to all drugs tested, other species, such as *S. epidermidis* and *S. haemolyticus*, exhibited resistance to clindamycin and cefoxitin, indicating potential failures in first-line therapies. Amikacin showed limited efficacy without prior sensitivity testing. These findings reinforce the need to personalize treatments based on laboratory data, avoiding the indiscriminate use of antimicrobials and reducing the risk of resistance.

### qPCR reaction

The analysis of the results of the 20 bacterial samples revealed that the positivity for Plasmid (P) genes was 65 %, while for Chromosomal (C) genes it was 35 %. This difference highlights the relevance of mobile genetic elements in the dissemination of antimicrobial resistance genes. Plasmids are known to facilitate horizontal gene transfer, contributing to the rapid dissemination of resistance in different environments, especially hospitals. The distribution of samples is shown in Table 6.

**Table 6:** Distribution of positive samples by genetic location.

Genetic Location	N° of Positive Samples	Percentage ( %)
Plasmidial (P)	13	65 %
Cromossomal (C)	7	35 %
Total	20	100 %

The samples presented information on different bacterial strains and the results of the detection of the *bla*Z, *fem*A and *mec*A genes, which are genetic markers associated with antimicrobial resistance in staphylococci. The *bla*Z gene was detected in 15 of the 20 samples, representing a prevalence of 75 %. This gene is associated with resistance to beta-lactams, highlighting its relevance in the use of penicillins.

The *fem*A gene was identified in 15 of the 20 samples, corresponding to 75 % of the samples. This gene is a marker for *Staphylococcus aureus* and is essential for cell wall biosynthesis. The mecA gene was detected in four of the 20 samples, corresponding to 20 % of the samples. This gene is associated with Methicillin Resistance (MRSA), representing a critical challenge in antimicrobial therapy.

When analyzing the distribution of resistance genes in the isolates studied, among the 20 *Staphylococcus* spp. samples studied, 75 % were positive for the femA gene, 80 % were positive for the *blaZ* gene and 15 % were positive for the mecA gene.

## Discussion

*Staphylococcus* spp. co-infections represent a significant therapeutic challenge. In neonatal patients, these infections are associated with higher mortality rates, prolonged hospitalization, and high healthcare costs.^14^ *S. lugdunensis*, which was identified in 25 % of the samples analyzed in this study, is of particular concern due to its ability to cause invasive infections similar to *S. aureus*.^15^

Global post-pandemic epidemiological reports indicate an increase in the prevalence of *Staphylococcus* spp. as co-infection agents in ICUs. An example to be cited is India, where studies reported that *S. aureus* co-infections were present in 25 %‒30 % of neonatal sepsis cases during the pandemic, with multidrug resistance rates exceeding 50 %.^16^

All isolates tested positive for the catalase test, a typical characteristic of the genus *Staphylococcus* spp. However, an atypical phenotypic profile was observed in *Staphylococcus aureus*, with gamma-hemolytic and coagulase-negative characteristics, diverging from the classic coagulase-positive and beta-hemolysis profile traditionally associated with this species.^17^^,^^18^

This result is particularly significant since coagulase is considered a crucial virulence factor in *S. aureus*, playing a central role in pathogenesis by facilitating the formation of blood clots, which helps the bacteria to evade the host's immune system.^19^ Although such characteristics are uncommon, phenotypic variations in *S. aureus* have been documented in response to environmental stresses, such as biofilm formation or adaptation to chronic infections, which can modulate the expression of virulence factors, including coagulase and hemolysis.^20^^,^^21^

Phenotypic variation in *Staphylococcus aureus* may have significant clinical implications, especially in chronic infections associated with biofilm formation. Although *S. aureus* is classically coagulase-positive, its ability to adapt to different environmental conditions may influence the efficacy of conventional treatments, requiring differentiated therapeutic approaches. On the other hand, coagulase-negative *Staphylococcus* species, such as *S. epidermidis*, are frequently associated with biofilms on medical devices, highlighting the need for specialized monitoring and treatment for these infections.^17^^,^^22^

There is a need for broader and more accurate diagnostic techniques to identify and treat phenotypic variations of *S. aureus*, thus reinforcing the importance of innovative and personalized therapeutic approaches to combat infections by atypical strains.

At the same time, *S. lugdunensis* presented beta-hemolysis and was coagulase-positive, characteristics that highlight its pathogenic potential and bring it closer to *S. aureus*. According to Babu et al.,^23^ serious infections caused by strains of *Staphylococcus lugdunensis* that present high virulence and antibiotic resistance have been documented. Similar to this, Oliveira et al.^24^ discusses the phenotypic variability in clinical strains of *Staphylococcus lugdunensis*, reinforcing the need for accurate diagnostic approaches. The presence of these characteristics implies important clinical considerations.

Coagulase-positive strains of *S. lugdunensis* may be less susceptible to conventional treatments and require a different therapeutic approach. This highlights the importance of monitoring and treating infections associated with biofilms, which may be relevant for infections caused by *S. lugdunensis*.^25^

Therefore, the results not only confirm the phenotypic variability of *Staphylococcus lugdunensis*, but also reinforce the need for broad and accurate diagnostic techniques to identify and treat phenotypic variations.

Other species, such as *Staphylococcus epidermidis*, which presented beta-hemolysis and positive coagulase, and *Staphylococcus haemolyticus*, with alpha-hemolysis and negative coagulase, exhibited distinct phenotypic profiles, which were essential for the specific identification of each isolate. These findings are in line with studies documenting phenotypic variability within the genus *Staphylococcus* spp., highlighting the importance of these differences for species identification and characterization.^17^^,^^26^

The manifestation of unexpected phenotypic characteristics, such as hemolysis and coagulase production in certain strains, may be associated with environmental adaptations or specific selective pressures. In this sense, Oliveira et al.^24^ reinforce the importance of understanding these phenotypic variations to elucidate the survival strategies and pathogenicity mechanisms of the different species of *Staphylococcus* spp.

In addition, *S. epidermidis* and S. *haemolyticus* are coagulase-negative and constitute the group of Coagulase-Negative Staphylococci (CNS), presenting significant antimicrobial resistance, becoming relevant in hospital infections. *S. lugdunensis* shares pathogenic characteristics with *S. aureus*, being capable of causing invasive infections. Furthermore, the formation of biofilm, a common characteristic of *S. epidermidis* and other coagulase-negative staphylococci, makes treatment difficult, since the bacteria in the biofilm are highly resistant to the action of antimicrobials and the host's immune response.^20^

The group is diverse in terms of virulence and antimicrobial resistance profile, and these results should be considered important, due to the diversity of patients. This detailed phenotypic profile provides.

Other tests are biochemical. They allow analysis of the microorganisms' ability to use certain substrates and produce certain metabolic products. They serve as a complement to identification based on phenotyping.^27^^,^^28^

Among these tests, the Novobiocin test stands out, a laboratory procedure used to identify different species of the genus *Staphylococcus* spp. This method can be used to determine whether a bacterium is sensitive or resistant to novobiocin, a type of antibiotic. When a bacterium is resistant to novobiocin, its growth continues close to the disk impregnated with the antibiotic, with little or no inhibition halo, indicating that the bacterium was not affected and can proliferate in the presence of the drug.^27^^,^^28^

On the other hand, when a bacterium is sensitive, a clear and well-defined halo forms around the disk, which demonstrates that novobiocin was effective in inhibiting bacterial growth. This analysis is especially essential for differentiating *Staphylococcus saprophyticus*, which is normally resistant to novobiocin, from other species, such as *Staphylococcus epidermidis*, which is usually sensitive to this antibiotic.^27^^,^^29^^,^^30^

The chosen antimicrobials stood out as good options for *Staphylococcus* spp. in the antibiogram, due to their coverage against resistance, diversity in mechanisms of action and their clinical use. These factors offered the possibility of effective coverage against strains of *Staphylococcus* spp., including those Resistant to Common Antibiotics Such as Methicillin (MRSA). Combined with the diversity of mechanisms of action, this allows testing of different targets within the bacterial cell, proteins, RNA, and cell wall, thus increasing the chances of identification for more effective clinical choices. Finally, these antimicrobials are widely used in clinical settings, especially in hospital or complex infections, which makes them relevant in the context of antibiograms.^31^

It was found a high level of resistance and this high level of resistance may indicate that a substantial number of bacterial infections may not respond to conventional treatments with Amikacin, resulting in a potential increase in serious and difficulty to treat infections. According to Ventola,^32^ antimicrobial resistance reduces the effectiveness of standard treatments, prolongs the duration of diseases, increases mortality and increases health care costs. In addition, the inappropriate and excessive use of antibiotics contributes to the rapid spread of resistant strains.^33^ These factors emphasize the urgent need for global strategies for the rational use of antibiotics and the development of new antimicrobial therapies.^34^

Regarding resistance to Cefoxitin in *Staphylococcus* spp., where 45 % of isolates were resistant, it reflects a worrying scenario in the control of bacterial infections, especially during the pandemic. The intensive use of antibiotics in hospitalized patients with COVID-19, often as a preventive measure against co-infections, may have contributed significantly to the selection of resistant strains. This phenomenon has been widely documented, indicating an increase in antimicrobial resistance in several pathogens due to selective pressure.^34^ In addition, the overloaded hospital environment during the pandemic increased the chances of dissemination of resistant strains of *Staphylococcus* spp., exacerbating the problem.^35^

Resistance of the isolates was also observed in clindamycin, rifampin and levofloxacin, where 25 %, 10 % and 20 %, respectively, were resistant. These data can be attributed to the intensive and sometimes indiscriminate use of antibiotics during the pandemic to treat secondary infections in hospitalized patients, which potentially favored the selection of resistant strains.^36^

Intermediate resistance was observed only for Levofloxacin, where 10 isolates (50 %) were intermediate. There was no intermediate resistance for the other classes of antimicrobials. The intermediate resistance observed in 50 % of *Staphylococcus* spp. isolates to levofloxacin highlights the complexity of the problem, suggesting that some strains may evolve to complete resistance if adequate measures are not taken.^37^

Regarding the sensitivity of the samples, 16 isolates, approximately 80 %, were sensitive to rifampicin, 65 % of the isolates, 13 samples were sensitive to clindamycin. Finally, it was observed that 55 %, 11 samples were sensitive to Cefoxitin, 6 samples, approximately 30 %, were sensitive to Amikacin and 6 isolates, approximately 30 % were sensitive to Levofloxacin.

These findings emphasize the need for integrated strategies to monitor and control multidrug resistance, especially in scenarios of co-infection with respiratory viruses such as COVID-19. This demonstrates the importance of monitoring antimicrobial resistance in *Staphylococcus* spp. infections.^38^

In Brazil, health surveillance data have also shown an increase in the rates of *Staphylococcus* spp. infections in hospitalized patients, especially in neonatal ICUs. The local scenario reflects the need for joint efforts to control the spread of resistant strains.^39^

According to Tacconelli et al.,^40^ the implementation of prevention programs in ICUs stands out, significantly reducing the prevalence of MRSA in hospitals that faced outbreaks during the pandemic. Optimizing the use of antimicrobials to promote the Antimicrobial Control Program (ACP) that prevents the selection of MDR strains, implementing active surveillance for early detection of resistant *Staphylococcus* spp., and investing in technologies for hospital decontamination, such as antimicrobial surfaces and monitoring of biofilms on medical devices, have reduced this prevalence.

During and after the COVID-19 pandemic, the epidemiological network in Brazil, at the national level, faced significant challenges related to antimicrobial resistance. The WHO warned that the indiscriminate use of antibiotics without confirmation of bacterial co-infections, such as *Staphylococcus* spp., contributed to the increase in microbial resistance, resulting in the emergence of MDR bacteria.^41^ In addition, co-infection with *Staphylococcus* spp., especially in patients hospitalized with COVID-19, increased the risk of serious infections, making treatments difficult.^42^

The results suggest that both global and local post-pandemic data reinforce the relevance of *Staphylococcus* spp. as priority agents in bacterial co-infections. The need for continuous surveillance, combined with serious, implementable, practical and effective control and prevention policies, is crucial to mitigate the impacts of co-infections in vulnerable populations, especially in neonatal ICUs.

Antimicrobial resistance has become a central focus in epidemiological surveillance, highlighting the need for a coordinated approach to monitor and control multidrug-resistant microorganisms.^33^

The presence of genes on plasmids, especially those related to antimicrobial resistance, is a concern in clinical settings, as it contributes to the rapid adaptation and evolution of pathogenic microorganisms. Recent studies, such as that of Partridge et al.,^43^ highlight that plasmids play an essential role in the global dissemination of resistance genes, such as *blaZ* and *mecA*.

On the other hand, the presence of chromosomal genes, although less prevalent, indicates stable resistance, fixed in the bacterial genome. This suggests that these bacteria may present intrinsic resistance and do not depend on mobile elements, representing specific challenges in the complete eradication of these strains in hospital and community settings.^44^

The presence of the *mec*A and *bla*Z genes in the plasmid samples suggests potential for horizontal spread of resistance, increasing the challenge in controlling infections. These findings reinforce the importance of monitoring and adapting treatment strategies according to the resistance profiles of the isolated strains.^45^

According to Kristich et al.,^46^ the prevalence of resistance genes is influenced by factors such as selective pressure caused by the indiscriminate use of antimicrobials and plasmid-mediated genetic mobility. The data in Table 6 corroborate this statement, especially in the context of the global increase in resistance rates reported in surveillance systems such as GLASS.^47^

## Conclusions

Therefore, the results reinforce the need to implement control and prevention strategies, including the rational use of antimicrobials and continuous monitoring of resistance rates. In addition, it is essential to promote additional studies to better understand the mechanisms of dissemination of resistance genes in clinical and community settings.

The widespread resistance observed in species such as *S. lugdunensis* (resistant to all antibiotics tested) suggests the emergence of complex molecular mechanisms, possibly linked to resistance genes or evolutionary adaptations. Atypical phenotypic and morphological variations in isolates also point to the circulation of less common strains, which may escape conventional detection methods. These data highlight the urgency of complementary genomic studies to elucidate markers of resistance and guide the development of new therapies.

The integration of laboratory data with the post-pandemic epidemiological scenario revealed a worrying increase in infections by *Staphylococcus* spp. in hospital settings, especially in ICUs. Species such as *S. aureus* and *S. epidermidis* were associated with medical devices and biofilms, while widespread resistance in *S. lugdunensis* reflects challenges in controlling critical infections. This evidence reinforces the need for continuous surveillance, biofilm prevention policies and investment in new drugs, aligning clinical and epidemiological strategies to mitigate impacts on public health.

The interaction between accurate diagnosis, resistance monitoring and epidemiological analysis is essential to address the complexity of *Staphylococcus* spp. coinfections. Phenotypic and molecular diversity, coupled with increasing resistance, requires integrated approaches and constant updating of protocols, ensuring effective treatments and reducing risks in critical clinical settings.

## Funding

1. FAPEG: Public called: 21/2024 Programa de Auxílio à Pesquisa Científica e Tecnológica Edição 2024. PROJECT: Characterization of Multidrug-Resistant Bacteria in Respiratory Virus Co-Infection in Children, coordinated by Doctor and Professor Lilian Carla Carneiro. 2. Called CNPq Nº 09/2022. CNPq: Productivity grant to Lilian Carla Carneiro (bench fee - 2023-2026).

## Conflicts of interest

The authors declare that they have no known competing financial interests or personal relationships that could have appeared to influence the work reported in this paper.

The authors declare the following financial interests/personal relationships which may be considered as potential competing interests:
